# Supplementary material for: Unveiling the Bioactive Potential of the Invasive Jellyfish Phyllorhiza punctata Through Integrative Transcriptomic and Proteomic Analyses
Source: Biomolecules. 2025 Aug 4;15(8):1121. doi: 10.3390/biom15081121 (PMC12383608; doi:10.3390/biom15081121)
Supplement: Supplementary file 1 [file biomolecules-15-01121-s001.zip › Suplementary Material/FileS3/PEPFOLD4/XP_065060543_4196/clusters.html]

 PEP-FOLD clusters


Download archive of all models  here   

|  |  |  |
| --- | --- | --- |
| # | file | sOPEP |
| # cluster 1 - Size: 2 |  |  |
|  | XP\_065060543\_4196-model1 | -10.8869 |
|  | XP\_065060543\_4196-model1.1 | -10.8869 |
| # cluster 2 - Size: 16 |  |  |
|  | XP\_065060543\_4196-model2 | -5.48606 |
|  | XP\_065060543\_4196-model2.1 | -5.41774 |
|  | XP\_065060543\_4196-model2.2 | -5.32226 |
|  | XP\_065060543\_4196-model2.3 | -5.27899 |
|  | XP\_065060543\_4196-model2.4 | -5.26951 |
|  | XP\_065060543\_4196-model2.5 | -5.26538 |
|  | XP\_065060543\_4196-model2.6 | -5.2567 |
|  | XP\_065060543\_4196-model2.7 | -5.25422 |
|  | XP\_065060543\_4196-model2.8 | -5.20872 |
|  | XP\_065060543\_4196-model2.9 | -5.20402 |
|  | XP\_065060543\_4196-model2.10 | -5.20099 |
|  | XP\_065060543\_4196-model2.11 | -5.17881 |
|  | XP\_065060543\_4196-model2.12 | -5.17581 |
|  | XP\_065060543\_4196-model2.13 | -5.15873 |
|  | XP\_065060543\_4196-model2.14 | -5.15074 |
|  | XP\_065060543\_4196-model2.15 | -5.09531 |
| # cluster 3 - Size: 9 |  |  |
|  | XP\_065060543\_4196-model3 | -5.30963 |
|  | XP\_065060543\_4196-model3.1 | -5.30963 |
|  | XP\_065060543\_4196-model3.2 | -5.30963 |
|  | XP\_065060543\_4196-model3.3 | -5.20234 |
|  | XP\_065060543\_4196-model3.4 | -5.19875 |
|  | XP\_065060543\_4196-model3.5 | -5.19875 |
|  | XP\_065060543\_4196-model3.6 | -5.14461 |
|  | XP\_065060543\_4196-model3.7 | -5.10099 |
|  | XP\_065060543\_4196-model3.8 | -5.08232 |
| # cluster 4 - Size: 16 |  |  |
|  | XP\_065060543\_4196-model4 | -5.26643 |
|  | XP\_065060543\_4196-model4.1 | -5.26643 |
|  | XP\_065060543\_4196-model4.2 | -5.26643 |
|  | XP\_065060543\_4196-model4.3 | -5.18163 |
|  | XP\_065060543\_4196-model4.4 | -5.18163 |
|  | XP\_065060543\_4196-model4.5 | -5.18163 |
|  | XP\_065060543\_4196-model4.6 | -5.14604 |
|  | XP\_065060543\_4196-model4.7 | -5.14604 |
|  | XP\_065060543\_4196-model4.8 | -5.12507 |
|  | XP\_065060543\_4196-model4.9 | -5.12507 |
|  | XP\_065060543\_4196-model4.10 | -5.12507 |
|  | XP\_065060543\_4196-model4.11 | -5.12507 |
|  | XP\_065060543\_4196-model4.12 | -5.12507 |
|  | XP\_065060543\_4196-model4.13 | -5.1077 |
|  | XP\_065060543\_4196-model4.14 | -5.1077 |
|  | XP\_065060543\_4196-model4.15 | -5.1077 |
| # cluster 5 - Size: 49 |  |  |
|  | XP\_065060543\_4196-model5 | -5.21666 |
|  | XP\_065060543\_4196-model5.1 | -5.21666 |
|  | XP\_065060543\_4196-model5.2 | -5.19392 |
|  | XP\_065060543\_4196-model5.3 | -5.19392 |
|  | XP\_065060543\_4196-model5.4 | -5.18423 |
|  | XP\_065060543\_4196-model5.5 | -5.18423 |
|  | XP\_065060543\_4196-model5.6 | -5.18423 |
|  | XP\_065060543\_4196-model5.7 | -5.18423 |
|  | XP\_065060543\_4196-model5.8 | -5.18423 |
|  | XP\_065060543\_4196-model5.9 | -5.18423 |
|  | XP\_065060543\_4196-model5.10 | -5.18423 |
|  | XP\_065060543\_4196-model5.11 | -5.16492 |
|  | XP\_065060543\_4196-model5.12 | -5.16492 |
|  | XP\_065060543\_4196-model5.13 | -5.16492 |
|  | XP\_065060543\_4196-model5.14 | -5.16492 |
|  | XP\_065060543\_4196-model5.15 | -5.16492 |
|  | XP\_065060543\_4196-model5.16 | -5.16492 |
|  | XP\_065060543\_4196-model5.17 | -5.16492 |
|  | XP\_065060543\_4196-model5.18 | -5.16492 |
|  | XP\_065060543\_4196-model5.19 | -5.16492 |
|  | XP\_065060543\_4196-model5.20 | -5.16492 |
|  | XP\_065060543\_4196-model5.21 | -5.16492 |
|  | XP\_065060543\_4196-model5.22 | -5.16492 |
|  | XP\_065060543\_4196-model5.23 | -5.16492 |
|  | XP\_065060543\_4196-model5.24 | -5.16492 |
|  | XP\_065060543\_4196-model5.25 | -5.16492 |
|  | XP\_065060543\_4196-model5.26 | -5.16492 |
|  | XP\_065060543\_4196-model5.27 | -5.1519 |
|  | XP\_065060543\_4196-model5.28 | -5.1519 |
|  | XP\_065060543\_4196-model5.29 | -5.1418 |
|  | XP\_065060543\_4196-model5.30 | -5.1418 |
|  | XP\_065060543\_4196-model5.31 | -5.13535 |
|  | XP\_065060543\_4196-model5.32 | -5.13535 |
|  | XP\_065060543\_4196-model5.33 | -5.12287 |
|  | XP\_065060543\_4196-model5.34 | -5.12287 |
|  | XP\_065060543\_4196-model5.35 | -5.11145 |
|  | XP\_065060543\_4196-model5.36 | -5.11145 |
|  | XP\_065060543\_4196-model5.37 | -5.11145 |
|  | XP\_065060543\_4196-model5.38 | -5.11145 |
|  | XP\_065060543\_4196-model5.39 | -5.11145 |
|  | XP\_065060543\_4196-model5.40 | -5.11145 |
|  | XP\_065060543\_4196-model5.41 | -5.11145 |
|  | XP\_065060543\_4196-model5.42 | -5.11145 |
|  | XP\_065060543\_4196-model5.43 | -5.11145 |
|  | XP\_065060543\_4196-model5.44 | -5.11145 |
|  | XP\_065060543\_4196-model5.45 | -5.11145 |
|  | XP\_065060543\_4196-model5.46 | -5.10944 |
|  | XP\_065060543\_4196-model5.47 | -5.09914 |
|  | XP\_065060543\_4196-model5.48 | -5.08422 |
| # cluster 6 - Size: 1 |  |  |
|  | XP\_065060543\_4196-model6 | -5.17121 |
| # cluster 7 - Size: 2 |  |  |
|  | XP\_065060543\_4196-model7 | -5.12087 |
|  | XP\_065060543\_4196-model7.1 | -5.0923 |
| # cluster 8 - Size: 5 |  |  |
|  | XP\_065060543\_4196-model8 | -5.11348 |
|  | XP\_065060543\_4196-model8.1 | -5.09443 |
|  | XP\_065060543\_4196-model8.2 | -5.09443 |
|  | XP\_065060543\_4196-model8.3 | -5.09443 |
|  | XP\_065060543\_4196-model8.4 | -5.08352 |
|  |  |  |
